# Village Charities

**Published:** 1890-02-15

**Authors:** 


### Village Charities.

*(Continued from page 289.)*

### II.—OCCASIONAL GIFTS.

In last week's paper on this subject, so much was said of hard-and-fast rules for the distribution of charities—rules which, it was urged, should be adhered to through thick and thin—that some may have felt disposed to rebel, and to exclaim—Are we always to be tied down in this way? Are our benevolent impulses to be for ever chained and fettered —denied all freedom of action, all choice as to *protégés?*

As to charities, yes; as to presents, no. It is only fair to ourselves that, in the matter of little occasional gifts, we should be allowed more freedom of choice. Where relief is needed, our rules should be hard as adamant; we feel that the poor have a right to ask why one should be helped, and not another, and that to have no good answer ready is an embarrassment and a shame to us. But it would be no good thing for them to be encouraged to question all our actions. We have special relations, special intimacies, with some of the poor; and if private gifts to them roused jealous questioning among the others, we should be quite within our right to say: "Mrs. So and So is my friend. It was a pleasure to me to give her a present, and a pleasure to her to receive it from me; and I can no more let you ask me why I gave to her and not to others, than I would listen to my brothers, if they complained—as they would never dream of doing—that I gave a present to my sister, and nothing to them." For presents and charities are different things, and should be looked upon as such.

Still, it is sometimes well to restrain our love of making presents. A real friendship is often damaged, rather than strengthened, by a continual and one-sided present-giving. And as for such gifts as ladies are fond of bestowing upon their special favourites—old clothes, and so forth, they may really be considered to come under the head of "relief." Unless they are needed for part of the outfit of a girl going to service (than which no better use could be found for them), it would be better to organize a little sale, now and again, of new and second-hand clothes. The prices might be so low that the clothes would still be practically given away; and it should be understood that the proceeds would go to some useful object, say, the purchase of materials for a Winter Work Society. No jealousies or heartburnings would be stirred by such a plan; and a present had far better take the form of a book, a paintbox, a picture or a pair of curtains, a comfortable chair, some flower seeds, a fife or an accordion—something, in short, that cannot be called "relief," but which yet may bring brightness, comfort, or refinement into the cottage home. Or what of a Savings Bank book, with an initial entry of a shilling or two? A lady once told the present writer that she always gave her presents to servant girls in this form, and that it had been the means of starting many in provident ways. Again, for those who think that our country poor have little knowledge of what a nourishing diet really means, and live far too exclusively on white bread, tea, and beer, a useful present will be a pound or two of oatmeal, a packet of cocoa, or a cordial,—say, a bottle of gingerette, which might often keep off a chill, and yet prevent recourse to the universal panacea of the uneducated—the brandy-bottle. See at the same time that facilities are provided for the procuring of these things; and your present may be a far more useful one than the packet of tea, or the half-worn dress, which you at first thought of giving.

But to come back to our charities. Some of these must of course always take the time-honoured form of blankets, coals, and so forth. But wherever we have a free hand, we may seriously put to ourselves this question:—Is there any way in which I may use this money, not merely for the temporary, but for the permanent, help of the poor? A blanket is very good while it lasts; but an outfit which helps a boy or girl to a place, gives that boy or girl a start in life, and is beyond comparison the more useful gift of the two. Again, a little present which shall help a man to begin pig, chicken, or bee-keeping, may often be the means of starting him in a profitable little industry, and have the very opposite effect on his character that a mere gift of clothes or food too often has. The proffered setting of eggs, or swarm of bees, may give just the little fillip to the rather slow-going mind of the country labourer which will start him on the road of enterprise and self-help; while the knowledge that you are prepared to give or lend a little money for the beginning of pig-keeping, or the taking on of a strip more allotment, may help him to realise dreams which have been reluctantly relegated to the background of his mind, for want of any apparent means for carrying them into effect. A kid, again, has been known to prove a most useful present to a labourer's family; the cost was but trifling, and its usefulness as milk-giver to the family great.

To seek out such ways as these for using our charity-money may indeed be more trouble than the spending of it upon mere relief. But surely we must deem our trouble well spent when we reflect on the serious injury done to the poor by many of our (so-called) charities. This injury cannot but be grave, when it causes an earnest and experienced clergyman to exclaim: "Had I the choice of a parish with or without charities, I would choose the latter!"—so bitterly does he feel the jealousies and discontents, the thriftlessness and indolence, the want of self-reliance and self-respect, which these charities induce in his parishioners.

It is well to remember, too, that even looking at the matter in its material aspect alone, our charities may be of far less service than we think. We know that the formation of a special fund for the relief of the poor in our metropolis will attract would-be recipients from all parts of the country, so that the distress it is intended to relieve may even be aggravated by it. And it may very well be that the knowledge that there are large endowed charities, or specially liberal gentlefolks, in a certain village, attracts thither more labourers than there is really work for, and so lowers the rate of wages. How to prevent the transference from the poor to the rich of charities meant for the poor alone is one of the most difficult and complex problems that can be set before us. It is said that in a certain village in the south of England, where there are endowed charities to the amount of £500 a year, the sole result is—high rents; and whether this be an exaggeration or not, it is certain that, in various ways, our charities are in great part practically alienated from the poor, and go to fill the pockets of the rich or well-to-do. Many employers of labour, again, are quick to note the fact that when they give a man no work to do the charitable people in the place will not allow him to suffer; and so village affairs fall into an unnatural and unhealthy state, the labour market becoming overstocked, the farmers careless of their responsibilities, the labourers indolent, dependent, and pauperised, and—the charitable people paying to keep all this up!

Truly it may be a consolation to some country parsons of the present day, who find themselves in sore straits through the agricultural depression, to reflect that they are at least free from the temptation—a very real one to a warm-hearted and impulsive man—to relieve their parishioners, masters and men, of their natural responsibilities and duties. But where they have the means at their disposal, whether their own or other people's, let them remember that, so far as they are free to choose, or to exert their powers of persuasion, it will be the truest charity to spend the money entrusted to them in helping the poor to help themselves—starting them in little industries, or new walks of life; or to use it in providing the village with a good supply of wholesome water, a workmen's club-room, a recreation ground, or a branch of a safe benefit club, rather than fritter it away in the mere relief of want. This may seem to some a hard doctrine; but is it harder than the undeniable fact that the relief of distress often tends but to perpetuate and to increase it?

